# Clinical, Microbiological and Pathological Findings of *Mycobacterium ulcerans* Infection in Three Australian Possum Species

**DOI:** 10.1371/journal.pntd.0002666

**Published:** 2014-01-30

**Authors:** Carolyn R. O'Brien, Kathrine A. Handasyde, Jennifer Hibble, Caroline J. Lavender, Alistair R. Legione, Christina McCowan, Maria Globan, Anthony T. Mitchell, Helen E. McCracken, Paul D. R. Johnson, Janet A. M. Fyfe

**Affiliations:** 1 Faculty of Veterinary Science, The University of Melbourne, Parkville, Victoria, Australia; 2 Department of Zoology, The University of Melbourne, Parkville, Victoria, Australia; 3 Newhaven Veterinary Clinic, Phillip Island, Victoria, Australia; 4 WHO Collaborating Centre for Mycobacterium ulcerans (Western Pacific Region), Victorian Infectious Diseases Reference Laboratory, North Melbourne, Victoria, Australia; 5 Department of Environment and Primary Industries, Veterinary Diagnostic Services, Bundoora, Victoria, Australia; 6 The University of Melbourne Veterinary Hospital, Werribee, Victoria, Australia; 7 Department of Environment and Primary Industries, Orbost, Victoria, Australia; 8 Melbourne Zoo, Zoos Victoria, Parkville, Victoria, Australia; 9 Department of Infectious Diseases, Austin Health, Heidelberg, Victoria, Australia; Fondation Raoul Follereau, France

## Abstract

**Background:**

Buruli ulcer (BU) is a skin disease caused by *Mycobacterium ulcerans*, with endemicity predominantly in sub-Saharan Africa and south-eastern Australia. The mode of transmission and the environmental reservoir(s) of the bacterium and remain elusive. Real-time PCR investigations have detected *M. ulcerans* DNA in a variety of Australian environmental samples, including the faeces of native possums with and without clinical evidence of infection. This report seeks to expand on previously published findings by the authors' investigative group with regards to clinical and subclinical disease in selected wild possum species in BU-endemic areas of Victoria, Australia.

**Methodology/Principal Findings:**

Twenty-seven clinical cases of *M. ulcerans* infection in free-ranging possums from southeastern Australia were identified retrospectively and prospectively between 1998–2011. Common ringtail possums (*Pseudocheirus peregrinus*), a common brushtail possum (*Trichosurus vulpecula*) and a mountain brushtail possum (*Trichosurus cunninghami*) were included in the clinically affected cohort. Most clinically apparent cases were adults with solitary or multiple ulcerative cutaneous lesions, generally confined to the face, limbs and/or tail. The disease was minor and self-limiting in the case of both *Trichosurus* spp. possums. In contrast, many of the common ringtail possums had cutaneous disease involving disparate anatomical sites, and in four cases there was evidence of systemic disease at post mortem examination. Where tested using real-time PCR targeted at IS*2404*, animals typically had significant levels of *M. ulcerans* DNA throughout the gut and/or faeces. A further 12 possums without cutaneous lesions were found to have PCR-positive gut contents and/or faeces (subclinical cases), and in one of these the organism was cultured from liver tissue. Comparisons were made between clinically and subclinically affected possums, and 61 PCR-negative, non-affected individuals, with regards to disease category and the categorical variables of species (common ringtail possums *v* others) and sex. Animals with clinical lesions were significantly more likely to be male common ringtail possums.

**Conclusions/Significance:**

There is significant disease burden in common ringtail possums (especially males) in some areas of Victoria endemic for *M. ulcerans* disease. The natural history of the disease generally remains unknown, however it appears that some mildly affected common brushtail and mountain brushtail possums can spontaneously overcome the infection, whereas some severely affected animals, especially common ringtail possums, may become systemically, and potentially fatally affected. Subclinical gut carriage of *M. ulcerans* DNA in possums is quite common and in some common brushtail and mountain brushtail possums this is transient. Further work is required to determine whether *M. ulcerans* infection poses a potential threat to possum populations, and whether these animals are acting as environmental reservoirs in certain geographical areas.

## Author Summary

*Mycobacterium ulcerans* causes skin disease predominantly in sub-Saharan Africa and southeastern Australia. The mode of transmission and the environmental reservoir(s) of the bacterium are unknown. Investigations have detected *M. ulcerans* DNA in a variety of Australian environmental samples, including the faeces of native possums. This report expands on these studies by detailing the clinical, pathological and microbiological findings in affected wild possum species in endemic areas. Twenty-seven clinically and 12 subclinically affected individuals were identified. Most clinical cases were adults with skin ulcers of the face, limbs and/or tail. The disease was mild and self-limiting in both *Trichosurus* spp. possums. In contrast, many of the common ringtail possums had multiple skin ulcers and in some there was evidence of internal disease. There were also significant levels of *M. ulcerans* DNA throughout the gut. Comparisons were made with regards to disease category, species and sex; with clinical cases more likely to be male common ringtail possums. Asymptomatic gut carriage of *M. ulcerans* DNA is quite common and may be transient in some individuals. Further work is needed to determine whether *M. ulcerans* infection poses a potential threat to possum populations, and whether these animals are acting as reservoirs in some areas.

## Introduction

*Mycobacterium ulcerans* is an environmental organism that causes distinctive dermal lesions in people and animals, via the elaboration of the cytotoxic and immunosuppressive polyketide toxin, mycolactone [1]. The disease is known internationally as Buruli Ulcer (BU). It has a worldwide but highly focal distribution, with endemicity recorded in 33 countries to date, predominately in sub-Saharan Africa and Australia. Sporadic cases and a number of localised outbreaks have been documented in people [2]–[13] and a variety of wild and domestic mammals [14]–[22] in southeastern Victoria, Australia, since the first reports of the disease over 60 years ago [3], [23].

Shortly after the initial reports of infection in people, it was shown that the common brushtail (CBT) possum (*Trichosurus vulpecula*, Kerr 1792) was experimentally susceptible to the disease [24], [25]. These investigations were undertaken because this species was noted to be highly susceptible to infection caused by members of the *M. tuberculosis* complex [26]. In brief, ulcerative lesions could be induced by local subcutaneous inoculation of organisms, and two animals inoculated via the intraperitoneal route developed lesions containing acid-fast bacilli (AFB) at peripheral sites (tail or tail base, scrotum, inguinal lymph node, stifle and front paw).

Despite this apparent susceptibility, there was no evidence of the infection in the wild CBT possum population in an endemic area of Victoria in the decades immediately following the initial outbreak, despite ongoing sporadic disease in the human population [3]. However, following an outbreak of the disease in people on Phillip Island, Victoria in the mid 1990s, two local wild common ringtail (CRT) possums (*Pseudocheirus peregrinus*, Boddaert 1785) were confirmed with *M. ulcerans* infection [18].

Since 2000, there has been an ongoing outbreak of BU in residents and visitors to the Bellarine Peninsula of Victoria [19]. Analysis of environmental samples using a highly sensitive real-time polymerase chain reaction (PCR) technique targeting sequences within IS*2404*, IS*2606* and KR [19], [27], [28] has confirmed low levels of *M. ulcerans* DNA in plant biofilms, vegetation, soil samples and local mosquitoes [29], and much higher levels (in some instances greater than 10^6^ organisms/gram), in the faeces of local wild CRT and CBT possums [19]. Despite this, the culture of viable organisms from these samples has remained elusive [19]. Variable-number of tandem repeats (VNTR) profiles of the DNA extracted from Point Lonsdale possum faeces was indistinguishable from that of the Victorian human outbreak strain of *M. ulcerans* [19], [28]. This report expands on the previous work performed by the authors' investigative group, by describing in detail the clinical, microbiological and pathological features of *M. ulcerans* infection of 27 clinical and 12 subclinical possum cases domiciled in three endemic areas of southern Victoria, Australia: Cowes (Phillip Island), Bellbird Creek (East Gippsland) and Point Lonsdale (Bellarine peninsula). This report represents the largest case series of naturally occurring *M. ulcerans* infection in any non-human species to date. Possible links between the disease in people and the resident possum population, and future avenues of investigation are discussed.

## Materials and Methods

### Case definitions

‘Clinical’ cases were defined as any possum with ulcerative skin lesions from which *M. ulcerans* was cultured, or was detected by IS*2404* PCR from clinical material, including swabs and necropsy tissues. ‘Subclinical’ cases were defined as any possum from which *M. ulcerans* was cultured or was detected by PCR from clinical material, including gut contents and/or faeces, where there was no gross or histopathological evidence of disease. During the course of the investigation, any possum in which no *M. ulcerans* DNA could be detected via PCR in any clinical sample was classified as ‘non-affected’.

### Retrieval of cases

Cases were identified by a review of the record database of the Mycobacterium Reference Laboratory of the Victorian Infectious Diseases Reference Laboratory (VIDRL) from 1998 to 2011, and from prospective capture and sampling of possums indigenous to some known *M. ulcerans-*endemic areas of Victoria; Bellbird Creek, between 2007–2012, as part of a routine surveillance program conducted by the Department of Environment and Primary Industries, and Point Lonsdale between 2008–2010, as part of an epidemiological study of *M. ulcerans* disease in these species. The methods of the latter study have been published elsewhere [19]. Clinical and/or autopsy data were also retrieved from case records of Newhaven Veterinary Clinic, Phillip Island, Victoria, the veterinary department of Melbourne Zoo, Parkville, Victoria, the laboratory records of the University of Melbourne Veterinary Hospital, Werribee, Victoria and the Department of Environment and Primary Industries, Veterinary Diagnostic Services, Victoria. Where available, data such as species, sex, estimated age at diagnosis (juvenile/adult), year of diagnosis, geographical location of domicile, anatomical location and nature of lesions, and results of autopsy examination (including histopathology) were recorded. Fresh faeces from mountain brushtail (MBT) possums (*Trichosurus cunnighami*, Lindenmayer 1990) trapped at Bellbird Creek were collected from within or underneath traps. For some possums trapped in Point Lonsdale, one to two millilitres of blood was collected from the cephalic, medial saphenous, or tail vein and placed in a plain tube. The blood sample was centrifuged at 16,100 g (Eppendorf 5415D, Hamburg, Germany) for 2 minutes before the serum was separated and stored at −80°C, the pellet was used for IS*2404* PCR analysis. Urine was collected via percutaneous cystocentesis where possible. Buccal and nasal swabs were obtained from some individuals. Fresh faeces were collected from underneath traps or from holding bags; a cloacal swab was collected from individuals if no faeces were obtained. Details of the methods utilised in the capture and sampling of possums has been described previously [19]. The project was approved by the University of Melbourne Faculty of Veterinary Science Animal Experimentation Ethics Committee (project no. 0706769) and was carried out under a permit from the Victorian Department of Sustainability and Environment (DSE permit no. 10004406).

Apart from case 28, which was found deceased, animals that underwent autopsy examination were euthanased with an overdose of sodium pentobarbitone, and tissues collected were either fixed in 10% buffered formalin for histopathology or were submitted fresh for microbiology. Formalin-fixed tissues were then embedded into paraffin blocks and processed routinely prior to staining with haematoxylin-eosin and/or acid-fast staining with either Ziehl-Neelsen (ZN) stain or Wade's modification of the ZN stain [30].

### Microbiological methods

Initially, IS*2404* PCR was performed by the Microbiological Research Unit, Royal Children's Hospital (prior to 2002) and thence at VIDRL, utilising the methods published by Ross et al [31]. Since 2003, a multiplex real-time PCR assay has been used to test clinical and environmental samples for the presence of *M. ulcerans* DNA [27].

Culture of *M. ulcerans* from lesions and necropsy tissue was carried out after decontamination via incubating at room temperature for 15 minutes with either 2 or 4% sodium hydroxide, followed by neutralisation with 10% orthophosphoric acid. The samples were then centrifuged at 3082 g (Beckman Coulter Allegra X-22, Pasadena, California, USA) for 20 minutes and the resultant pellet was re-suspended in 2 ml of Ringer's solution. 400 µl was then used to inoculate Brown and Buckle slopes, and Mycobacterium Growth Indicator Tubes (BD, Franklin Lakes, N. J.) admixed with 0.8 ml of manufacturer-supplied antibiotic mixture and incubated for up to 12 weeks at 31°C, with weekly or fortnightly monitoring by either visual inspection or IS*2404* real-time PCR.

Isolates were typed by either restriction fragment length polymorphism (RFLP) typing [32], variable-number of tandem repeats (VNTR) typing [33], [34] and in two instances, whole genome sequencing [35].

### Statistical analysis

The two-tailed Fischer exact test was used to test for associations between categorical variables. The t-test was performed when comparing the mean of continuous variable data sets. P values<0.05 were considered significant.

## Results

### Trapping studies

#### Point Lonsdale

Numerous samples were collected from 69 possums.. Faecal samples were collected from 57 individual animals; in the first instance 14 (25%) of these samples were PCR-positive for *M. ulcerans* DNA. In six animals multiple faecal samples were collected over the course of the study; in three cases 2 of 2 samples were negative, in one case 2 of 2 were positive and in three cases 1 of 2, 1 of 5 and 1 of 6 samples were PCR-positive respectively. Blood samples were collected from 63 individuals, none of which were PCR-positive. Buccal swabs were obtained from 67 individuals; 7 (16%) were PCR-positive. Urine samples were collected from 16 animals; all were PCR-negative. Pouch swab samples were collected from 15 possums; 3 (20%) of which were weakly PCR-positive. Twenty cloacal swab samples were collected (often in lieu of a faecal sample); the one instance where this sample was PCR-positive, the animal was concurrently documented to have PCR-positive faeces.

#### Bellbird Creek

Sixty-nine faecal samples were collected from 15 MBT possums that were trapped (some repeatedly) from 2007–2012. Four animals (27%) had a PCR-positive faecal sample on a single occasion.

### Clinical cases

Twenty-seven clinically affected animals were identified for inclusion in the study (Table 1). There were 23 CRT, one CBT and one MBT possum. For two possums, species was not recorded. Of the 25 animals for which sex was recorded 17 (69%) were male. Of the 24 animals in which an age estimate was performed, one was a juvenile and 23 were estimated to be adults (although more objective data such as dental examination were not utilised).

**Table 1:** Data for possums with confirmed clinical *M. ulcerans* infection.

Case:	Domicile:	Yr diag:	Species:	Sex:	Est age:	Comments:
1	Phillip Island	1998	CRT	NR	Adult	Ulcers on nasal bridge and hind feet.
2	Phillip Island	2000	CRT	F	Adult	Ulcer on dorsal nasal bridge.
3	Phillip Island	2000	CRT	M	Adult	Ulcers on distal third of tail, lateral right tail, bilateral medial distal tibia, ventral tail, scrotum, medial left eye.
4	Phillip Island	2001	NR	M	NR	NR
5	Phillip Island	2001	CRT	M	Adult	Good BCS. Severe ulceration right side of the face and mandible, extending down to facial bone.
6	Phillip Island	2002	CRT	M	NR	NR
7	Phillip Island	2002	CRT	NR	Adult	Ulcer lateral face/jaw.
8	Phillip Island	2003	NR	M	Adult	Ulcer dorsal tail base and mid-tail.
9	Phillip Island	2003	CRT	F	Adult	Severe ulcerations on tail, both hind feet, carpi and dorsum of nose.
10	Bellbird Creek	2005	MBT	M	Adult	Ulcer on left hind foot.
11	Phillip Island	2007	CRT	M	Juvenile	Ulcers on nose and feet.
12	Point Lonsdale	2008	CRT	F	Adult	Poor BCS. Ulcers on tail tip and toe of right hind foot.
13	Point Lonsdale	2008	CRT	M	Adult	Numerous small ulcers on ventral tail.
14	Point Lonsdale	2008	CRT	M	Adult	Good BCS. Ulcer ventral tail. Euth. and full autopsy exam.
15	Point Lonsdale	2008	CRT	M	Adult	Good BCS. Ulcer dorsal tail. Euth. and full autopsy exam.
16	Point Lonsdale	2008	CRT	F	Adult	Fair BCS. Ulcers on nose and tail. Lip, face and front paws oedematous. Euth. and full autopsy exam.
17	Phillip Island	2008	CRT	M	Adult	Ulcers on face, hock and tail.
18	Phillip Island	2009	CRT	M	NR	Ulcer on ear.
19	Point Lonsdale	2009	CRT	M	Adult	Good BCS. Small ulcer on toe of front foot.
20	Point Lonsdale	2009	CBT	F	Adult	Good BCS. Small ulcer on left front toe. Lesion resolved when re-trapped 11 months later.
21	Point Lonsdale	2009	CRT	M	Adult	Good BCS. Small ulcer on left hind toe.
22	Point Lonsdale	2009	CRT	M	Adult	Good BCS. Oedematous ulcer on tail.
23	Point Lonsdale	2009	CRT	F	Adult	Good BCS. Large oedematous ulcer tail and small ulcer on ear.
24	Point Lonsdale	2009	CRT	M	Adult	Poor BCS. Oedematous ulcer ventral tail, nose, left antebrachium, right face. Euth. and full autopsy exam.
25	Point Lonsdale	2009	CRT	F	Adult	Poor BCS. Oedematous ulcer on tail. Left corneal opacity. Oedema left nostril and lips. Euth. and full autopsy exam.
26	Point Lonsdale	2010	CRT	M	Adult	Ulcer on tail. Radio collar fitted later found dead with large necrotic lesion on chest.
27	Phillip Island	2011	CRT	F	Adult	Poor BCS. Ulcers on right medial tibia and caudal hock, left medial tibia and caudal hock, bilateral fore caudal carpus and cranial antebrachium, ventral tail near base and tip. Euth. and autopsy exam

CRT = common ringtail possum, CBT = common brushtail possum, NR = not recorded, F = female, M = male, BCS = body condition score, Euth. = euthanasia.

**Figure 1:**
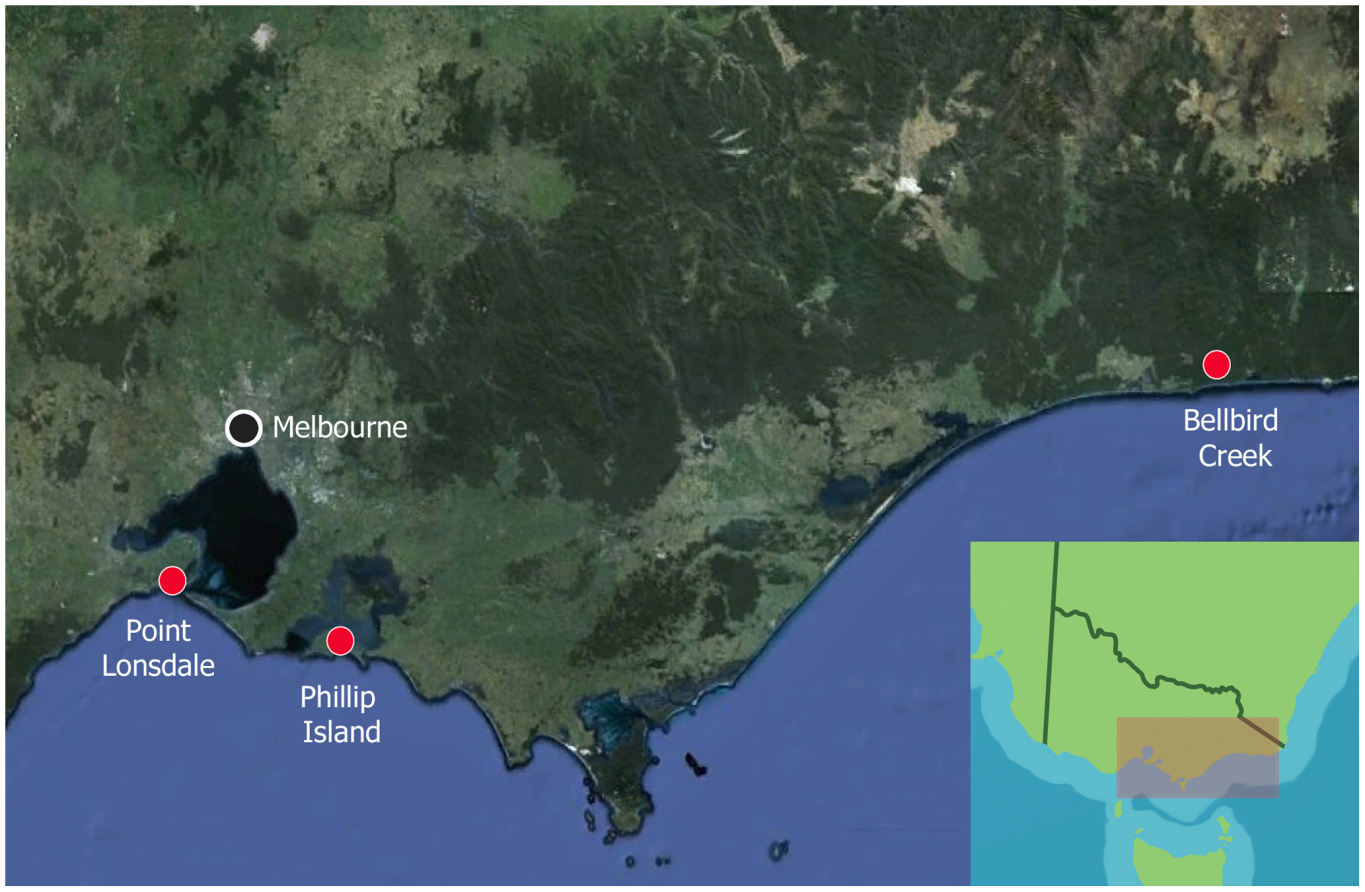
Map of south-eastern Victoria, showing the geographical location of possum cases (red dots).

Thirteen of the animals (11 CRT possums and two species not recorded) were from the Cowes region of Phillip Island, Victoria (Figure 1). These animals had been surrendered to the local veterinary hospital (Newhaven Veterinary Clinic) because of illness or trauma between 1998 and 2011. The MBT possum was captured in 2005 at Bellbird Creek (Figure 1). This animal was transferred to Melbourne Zoo, Parkville, Victoria, for management. Thirteen animals (12 CRT and one CBT) were from Point Lonsdale, Victoria (Figure 1); all were trapped between 2008–2010 as part of the prospective epidemiological study.

**Figure 2:**
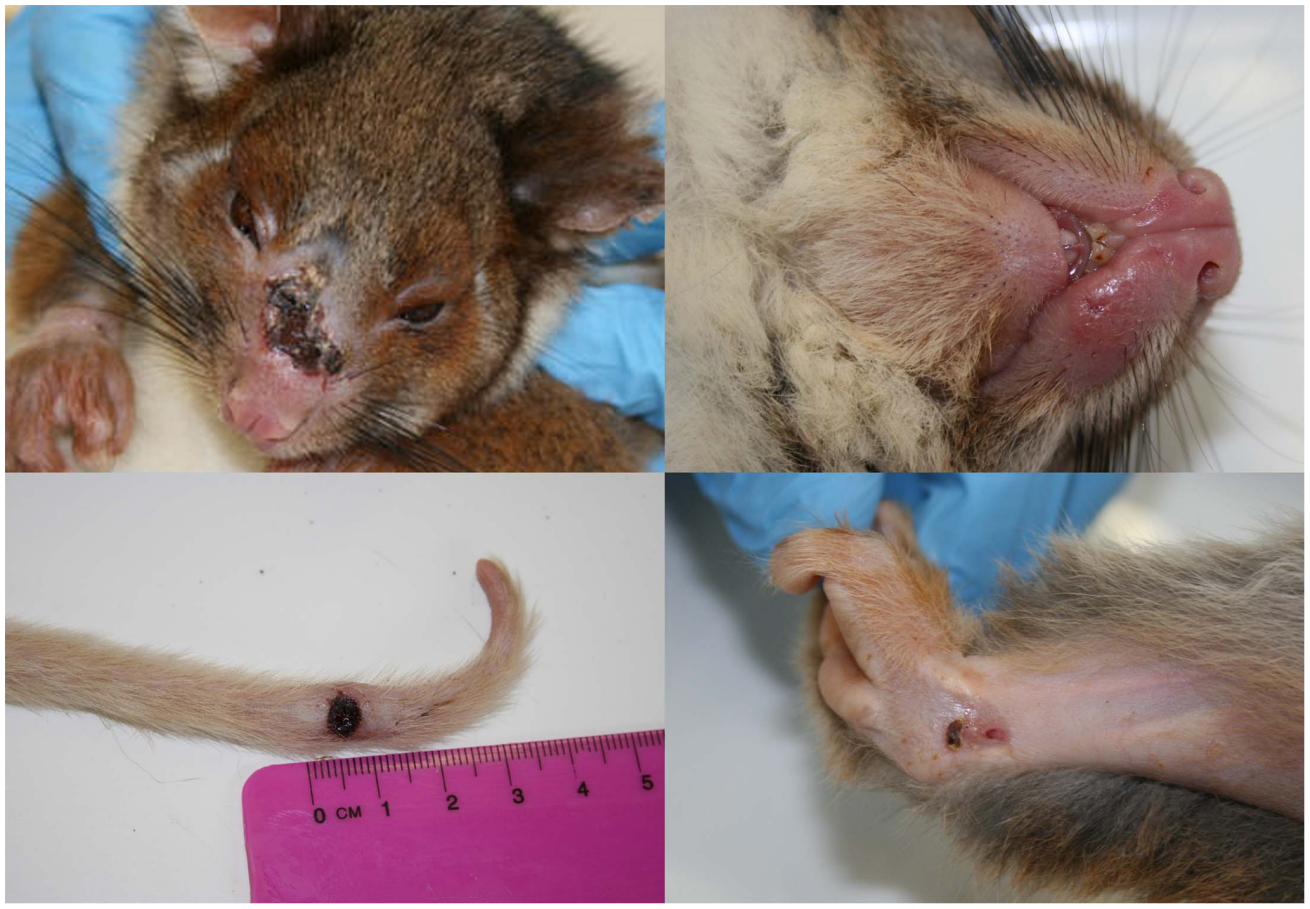
Lesions caused by *M. ulcerans* infection in a common ringtail possum (case 16). This animal had multiple affected sites characterised by ulceration and/or oedema. (Image C. McCowan/J. Fyfe).

**Figure 3:**
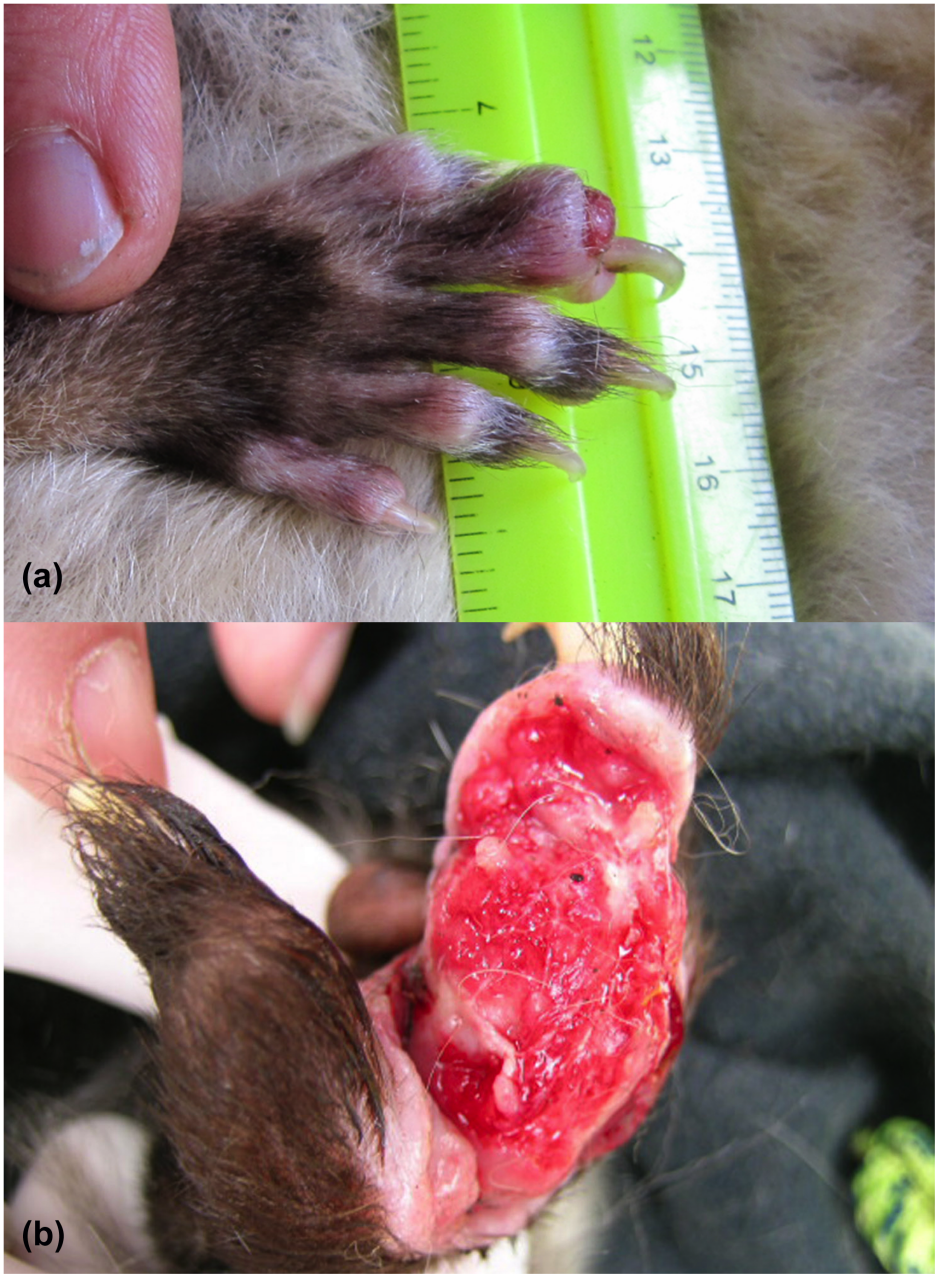
(a): Toe lesion in a common brushtail possum (case 20) (Image T. Ruf), (b): Ulceration caused by *M. ulcerans* on the left hind foot of a mountain brushtail possum (case 10). This lesion later underwent spontaneous resolution. (Image H. McCracken).

The nature and anatomical location of lesions were recorded for 25 possums (Table 1). Twelve animals had single skin lesions, five had two skin lesions and eight animals had multiple skin lesions, involving three or more anatomical sites. Lesions recorded on CRT possums were located on the face and/or head (n = 13), feet and/or limbs (n = 11) and tail and/or tail base (n = 14) (Figure 2). One animal also had scrotal ulcers (as well as multiple lesions at other sites). One CRT fitted with a radio-tracking collar (case 26), noted to initially have a solitary tail lesion, was found deceased several months later with a large IS*2404* PCR-positive lesion on the ventrolateral thorax. The CBT possum had a small ulcer on a front toe (Figure 3a) and the MBT had a single ulceration on the left hind foot (Figure 3b). Both of these animals were noted to have spontaneous healing of lesions without specific treatment over a 3–6 month period. Only CRT possums were recorded to have two or more lesions, with six disparate anatomical sites in two instances.

**Figure 4:**
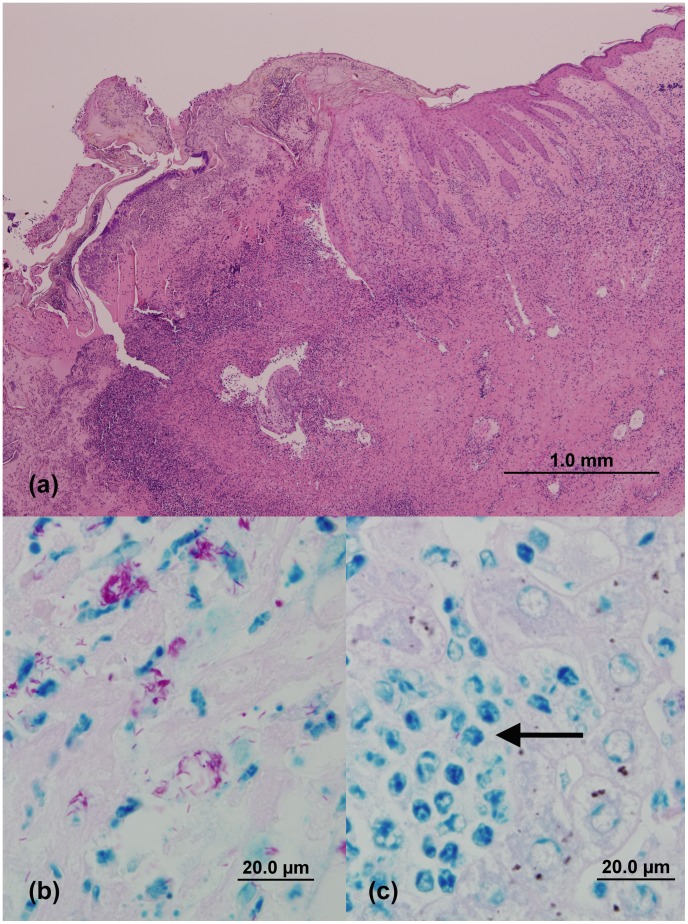
(a): Photomicrograph of a skin lesion obtained from case 16. The lesion is characterised by proliferative epidermis overlying fibrotic dermal tissue admixed with inflammatory cells, and superficial crust composed of serous exudate and degenerate leukocytes overlying a necrotic base. (H&E stain)) (**b**): Demonstration of numerous acid-fast bacilli (AFB) in an ulcerated skin lesion of case 16 (modified ZN stain), (**c**): the liver lesions in this case contained rare AFB, mostly within macrophages (arrow) (modified ZN stain). (Images C. McCowan).

Eight animals (cases 5, 8, 14–16, 24, 25, and 27) underwent partial or complete autopsy examination. The necrotic skin ulcers appeared grossly and histopathologically similar to those described in other species [16], [22], [36]. The lesions were characterised by extensive loss of the epidermis. A superficial crust composed of serous exudate and degenerate leukocytes was present, overlying a necrotic base (Figure 4a). The margins of the lesions were characterised by proliferative epidermis overlying fibrotic and sometimes, oedematous dermal tissue admixed with pyogranulomatous and/or lymphoplasmacytic inflammation. In ZN-stained sections, abundant numbers of AFB were present in the dermis, either extra-cellularly, or within macrophages (Figure 4b).

Four animals with extensive cutaneous disease (cases 16, 24, 25 and 27) also had evidence of systemic involvement, with patchy granulomatous, neutrophilic and/or lymphoplasmacytic lesions in the liver and lung. Rare AFB were present in macrophages in some sections of liver (Figure 4c).

The results of the microbiological investigations for clinically affected animals performed on either lesion swabs and/or necropsy material and, in most cases, gut contents or faeces are presented in Tables 2 and 3. *M. ulcerans* was cultured from skin lesions only in 19 animals, the liver, spleen and a mandibular lymph node in case 19, and skin lesions, liver, lung and small intestinal contents in case 27. The organism was also cultured from the abdominal cavity of the decomposing carcass of case 26.

**Table 2:** Microbiological data for possums with clinical *M. ulcerans* infection.

Case:	IS*2404* PCR results:	Culture results:	Typing results:
1	PCR +ve: nose lesion	Culture +ve: nose lesion	NA
2	PCR +ve: nose lesion	Culture +ve: nose lesion	NA
3	PCR +ve: site not specified	Culture +ve: site not specified	NA
4	PCR +ve: site not specified	Not cultured	NA
5	PCR +ve: lesion swab and stomach wall. PCR −ve: liver	Culture +ve: facial ulcer tissue. Culture −ve: liver and stomach wall	RFLP ‘V1’
6	PCR +ve: site not specified	Culture +ve: site not specified	RFLP ‘V1’nVNTR ‘Victorian’
7	PCR +ve: site not specified	Culture +ve: site not specified	RFLP ‘V1’ VNTR ‘Victorian’
8	PCR +ve: tail base and mid-tail lesions	Culture +ve: both tail lesions	RFLP ‘V1’
9	PCR +ve: site not specified	Culture contaminated	NA
10	PCR +ve: site not specified	Culture +ve: left hind foot lesion	VNTR ‘Victorian’ WGS
11	PCR +ve: site not specified	Culture +ve: site not specified	VNTR ‘Victorian’
12	PCR +ve: tail and toe lesions, buccal swab. PCR −ve: blood sample	Culture +ve: toe and tail lesions	VNTR ‘Victorian’
13	PCR +ve: tail lesions. PCR −ve blood, faeces, cloacal and nasal swab	Culture not performed	NA
14	PCR +ve: tail lesion, gut contents, faeces. PCR −ve: buccal and nasal swabs, blood, bile, urine, salivary gland, lung, liver, spleen, kidney	Culture +ve: tail lesion. Culture −ve: gut contents	VNTR ‘Victorian’
15	PCR +ve: tail lesion, faeces, salivary gland, gut contents. PCR −ve: buccal and nasal swabs, blood, bile, urine, lung, liver, spleen, kidney	Culture +ve: tail lesion. Culture −ve: salivary gland, gut contents	VNTR ‘Victorian’
16	PCR +ve: multiple limb lesions, facial lesions, tail lesion, lung, liver, spleen, kidney, mandibular lymph node, heart, mesenteric lymph node, salivary gland, buccal swab, gut contents	Culture +ve: liver, nose lesion, mandibular lymph node, spleen. Culture −ve: hind leg muscle, colon, right hock,stomach tissue, gut contents	VNTR ‘Victorian’ WGS
17	PCR +ve: from swab of multiple combined sites	Culture +ve: swab of multiple combined sites	VNTR ‘Victorian’
18	PCR +ve: ear tissue	Culture −ve: ear tissue	NA
19	PCR +ve: faeces, toe lesion. PCR −ve: blood, buccal and nasal swab	Culture not performed	NA
20	PCR +ve: toe lesion, faeces. PCR −ve: blood, buccal and pouch swabs	Culture +ve: toe lesion	VNTR ‘Victorian’
21	PCR +ve: toe lesion, faeces, buccal swab. PCR −ve: blood	Culture −ve: toe lesion	NA
22	PCR +ve: tail lesion, faeces, buccal swab. PCR −ve: blood	Culture +ve: tail lesion	VNTR ‘Victorian’
23	PCR +ve: pouch, buccal swab, nasal swab, tail and, ear lesion swabs, faeces. PCR −ve: blood	Culture not performed	NA
24	PCR +ve: faeces, tail,eye and nose lesions, buccal, and pouch swabs, spleen, lung, kidney, liver, gut contents	Culture +ve: tail lesion. Culture −ve: gut contents	VNTR ‘Victorian’
25	PCR +ve: eye lesion, nasal lesion, lung, liver, spleen, kidney, buccal swab, gut contents	Culture +ve: tail lesion. Culture −ve: gut contents	VNTR ‘Victorian’
26	PCR +ve: tail lesion, buccal. PCR −ve: clocal swab	Culture +ve: caudal abdominal cavity	VNTR ‘Victorian’
27	PCR +ve: tail and hind leg lesions, liver, lung, gut contents	Culture +ve: tail and hind leg lesions, liver, lung and SI contents. Culture −ve: stomach, caecum and LI contents	VNTR ‘Victorian’

IS*2404* PCR = polymerase chain reaction targeted against insertion sequence *2404*, +ve = positive, −ve = negative, NA = not available, RFLP = restriction fragment length polymorphism, VNTR = variable numbers tandem repeats, WGS =  whole genome sequencing, SI = small intestinal, LI = large intestinal, real-time PCR cycle threshold (C_T_): (+/−) = C_T_ >35, (+) = C_T_ >30–35, (++) = C_T_ >25–30, (+++) = C_T_ >20–25, (++++) C_T_≤20.

**Table 3:** Relative real-time IS*2404* PCR signal from gut contents of possums.

Case:	Stomach Contents:	SI Contents:	Caecal Contents:	LI Contents:
14	+	+	+/−	+
15	+/−	+	+	+/−
16	+++	++	++	++
24	+++	++	+++	+++
25	+++	++	+++	++
27	++	+	+++	++
28	++	++	NP	+
32	++	++	+	++

SI = small intestinal, LI = large intestinal, real-time PCR cycle threshold (C_T_): (+/−) = C_T_ >35, (+) = C_T_ >30–35, (++) = C_T_ >25–30, (+++) = C_T_ >20–25, (++++) C_T_ ≤20, NP = not performed.

RFLP typing of isolates was performed in four cases and found to be “V1” type [32]. VNTR typing was performed in 15 cases and was found to be indistinguishable from *M. ulcerans* strains causing human and animal disease in Victoria. Analyses of the whole genome sequences of the isolates from cases 10 and 16, which have been described previously [35], confirmed a close genetic relationship with *M. ulcerans* isolates from Victorian human patients.

The duodenum of case 16 also contained a large tapeworm (species not identified), which was PCR-negative, despite the moderate real-time PCR-positivity of the small-intestinal contents of this animal. PCR-positive fly larvae (species not identified) were also retrieved from the carcass of case 26, however *M. ulcerans*, could not be cultured successfully from these larvae.

### Subclinical cases

**Figure 5:**
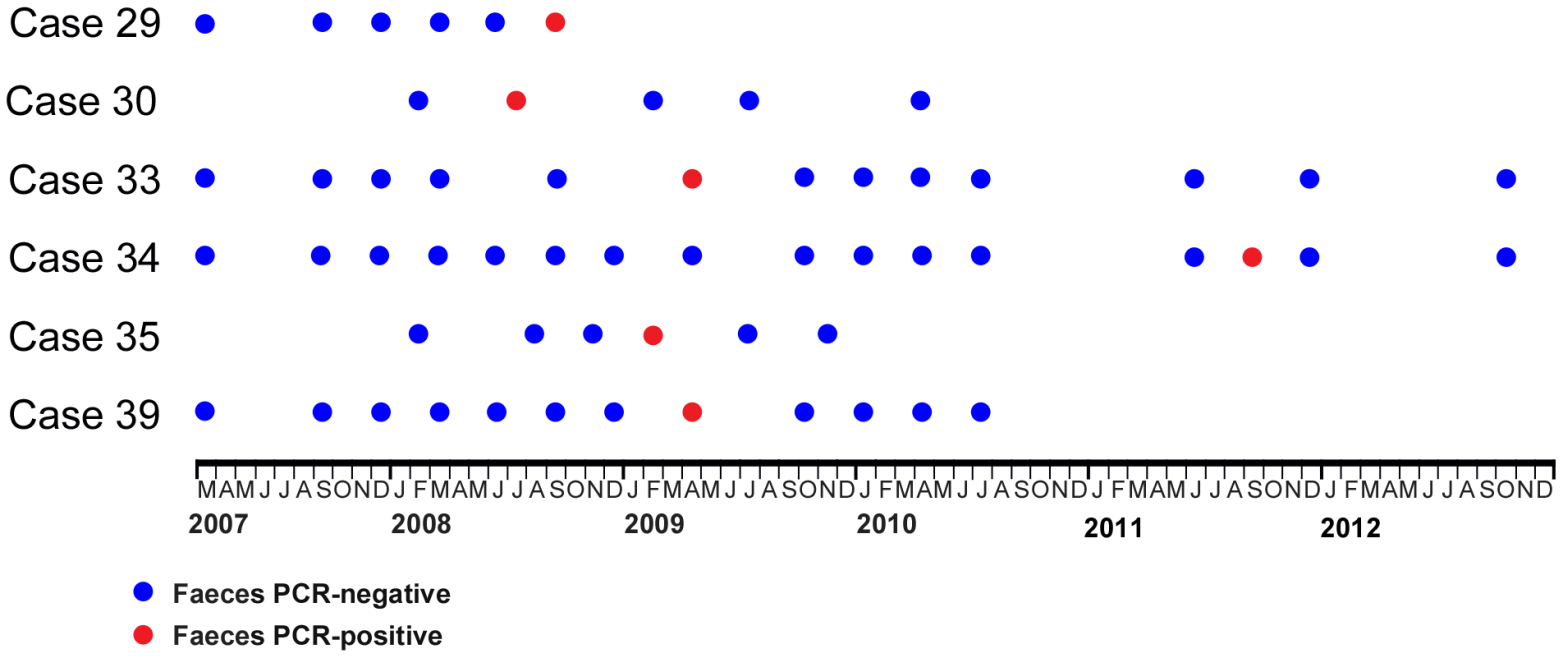
Graphical representation of PCR results of faecal samples collected from sub-clinically affected common brushtail and mountain brushtail possums that were repeatedly trapped during the period of the study. In the majority of these individuals the gut carriage of *M. ulcerans* DNA was demonstrated to be transient.

Twelve animals met the criteria for classification as a subclinical case (Table 4). These were all adult animals, comprising one male and three female CBT possums, four female CRT possums, and one male and three female MBT possums. The animals were domiciled in Point Lonsdale (CBT and CRT possums), and Bellbird Creek (MBT possums). One animal (case 28) was identified by a Point Lonsdale resident, while the remainder were trapped as part of the aforementioned studies (cases 29–39). Some CBT and MBT possums were trapped multiple times; the time-line of results from PCR analysis on faecal samples from these animals is presented in Figure 5.

**Table 4:** Data for sub-clinically affected possums.

Case :	Domicile:	Yr. diag.:	Species:	Sex:	Est. age:	Comments:
28	Point Lonsdale	2007	CBT	M	Adult	Found dead by resident. No external lesions or internal pathology at autopsy. Culture +ve from liver tissue.
29	Bellbird Creek	2008	MBT	F	Adult	No external lesions. PCR +ve faeces (++) on one trapping occasion.
30	Point Lonsdale	2008	CBT	F	Adult	No external lesions. PCR +ve faeces (+/−) on one trapping occasion. Blood, buccal swabs, nasal swabs and urine repeatedly PCR −ve.
31	Point Lonsdale	2008	CRT	F	Adult	Poor BCS. No external lesions. PCR +ve faeces (++)
32	Point Lonsdale	2008	CRT	F	Adult	No external or internal lesions on full autopsy. PCR +ve gut contents.
33	Bellbird Creek	2009	MBT	M	Adult	No external lesions. PCR +ve faeces (+/−) on one trapping occasion.
34	Bellbird Creek	2009	MBT	F	Adult	No external lesions. PCR +ve faeces (++) on one trapping occasion.
35	Point Lonsdale	2009	CBT	F	Adult	No external lesions. PCR +ve faeces (+) on one trapping occasion. Blood, buccal swabs and nasal swabs repeatedly PCR −ve.
36	Point Lonsdale	2009	CBT	F	Adult	No external lesions. PCR +ve faeces (+/−)
37	Point Lonsdale	2009	CRT	F	Adult	No external lesions. PCR +ve faeces (+/−)
38	Point Lonsdale	2009	CRT	F	Adult	No external lesions. PCR +ve faeces (+), buccal swab (+/−)
39	Bellbird Creek	2011	MBT	F	Adult	No external lesions. PCR +ve faeces (+/−) on one trapping occasion.

PCR = polymerase chain reaction targeted against insertion sequence IS*2404*, +ve = positive,

(+/−) = real-time PCR cycle threshold (C_T_) >35, (+) = C_T_ >30–35, (++) = C_T_ >25–30, (+++) = C_T_ >20–25, (++++) C_T_ ≤20.

[30] → Two subclinical cases (cases 28 and 32) underwent autopsy examination. Case 28 had been seen at ground level during the late afternoon and had appeared unusually docile. The animal was found deceased the following day and its body was submitted to the veterinary pathology service of The University of Melbourne a few days later. Although there was significant tissue autolysis, and cause of death could not be determined, there were no cutaneous lesions consistent with *M. ulcerans* infection. Low levels of *M. ulcerans* DNA were detected by real-time PCR in tissue from the liver, spleen, lung and gut wall, as well as small and large bowel contents and faecal pellets retrieved from the rectum. *M. ulcerans* as also cultured from the liver tissue. The carcass also contained fly larvae (species not identified), which were also weakly IS*2404* PCR-positive.

Case 32 was found to have IS*2404* PCR-positive faeces and was euthanased and underwent autopsy as part of the epidemiological study. The animal had no gross or histopathological lesions. A moderate level of *M. ulcerans* DNA was detected in all gut compartments (Table 3), and salivary gland tissue was also weakly PCR-positive [35].

### Non-affected possums

Of the 69 animals trapped in Point Lonsdale, 50 (72%) had no detectable *M. ulcerans* DNA (via IS*2404* real-time PCR) in any clinical sample. This cohort comprised 13 male and 6 female CBT possums, and 13 male and 18 female CRT possums. Eleven (73%) MBT possums trapped at Bellbird Creek had PCR-negative faecal samples on all trapping occasions. This group of animals comprised 7 males and 4 females.

### Comparison of clinically, sub-clinically and non-affected possums

Categorical variables of (i) case type (clinical, subclinical and non-affected) (ii) species (CRT possum and non-CRT possum), and (iii) sex (male and female) were compared using 2×2 contingency tables (raw data presented in Table 5).

**Table 5:** Numbers of common ringtail and non-common ringtail (common brushtail and mountain brushtail) possums categorised by sex and case category.

Case Category:	Male CRT	Female CRT	Sex unknown CRT	Male Non-CRT	Female Non-CRT	Male Unknown Species
**Clinical**	14	7	2	1	1	2
**Subclinical**	0	4	-	2	6	-
**Non-affected**	13	18	-	20	10	-

CRT = common ringtail possum.

A comparison of animals with (clinical) and without cutaneous lesions (subclinical and non-affected possums) was made; the former animals were more likely to be CRT possums, or male, although the latter variable did not reach significance. When the variables of sex and species were combined, male CRT possums were more likely to have clinical lesions than other animals (odds ratios, including 95% confidence intervals and P values are presented in Table 6).

**Table 6:** Statistical comparison of possums with (clinical cases) and without cutaneous lesions (subclinical and non-affected cases).

Variable:	Odds Ratio; 95% Confidence Intervals	P value
**Species (CRT)**	12.22; 2.696–114.5	0.0001
**Sex (M)**	2.288; 0.8126–6.941	0.1
**Species (CRT) + Sex (M)**	6.991; 2.278–22.89	0.0003

CRT = common ringtail possum, M = male.

When comparisons were made between animals with a PCR-positive sample (both clinical and subclinical cases) and those in which all clinical samples were PCR-negative (non-affected cases) (results presented in Table 7), individuals in the former group were more likely to be CRT possums. There was no sex predilection for PCR-positivity.

**Table 7:** Statistical comparison of possums with (clinical and subclinical cases) and without PCR-positive samples (non-affected cases).

Variable:	Odds Ratio; 95% Confidence Intervals	P value
**Species (CRT)**	2.587; 1.003–7.081	0.05
**Sex (M)**	0.8459; 0.3461–2.065	0.1

CRT = common ringtail possum, M = male.

The mean IS*2404* real-time PCR signal strength (C_T_) of approximately 100 mg of faeces collected from clinically (n = 10) and sub-clinically affected (n = 12) animals was calculated as 29.32 (SD ±5.43) and 33.54 (SD ±4.82), respectively (raw data not shown) (Figure S1). When the values were compared, the difference was not statistically significant (P = 0.075).

## Discussion

Although there are previous reports of *M. ulcerans* infection in possums [18], [19], this is the first study to examine the clinical, pathological and microbiological aspects of the disease in detail, and also represents the largest and most comprehensive case series of the disease in any non-human species. Affected possums have only been recorded from areas of known *M. ulcerans* endemicity in Victoria, Australia. This is true for all reports of the infection in non-human mammalian species, despite the fact that the disease has been reported in many disparate areas of the world. The reasons for this epidemiological discrepancy are currently unknown, but may be due to specific host or environmental factors (for example, the presence of particular insect vectors in a temperate climate), or genomic differences related to virulence or host specificity of the ‘Victorian’ strain of *M. ulcerans*, compared to strains found elsewhere. There has been some attempt to identify animal cases in endemic areas of Africa and other parts of Australia, however this research has not yet yielded any positive results (J. Fyfe, C. Lavender, PDR. Johnson unpublished observations) [37], [38].

It has been reported that some species of marsupials appear prone to particular mycobacterial diseases [39] (for example, CBT possums and *M. bovis* infection, macropods and infections caused by the *M. avium* complex) and it is noteworthy that the next animal species most commonly reported with *M. ulcerans* infection is another Australian arboreal marsupial, the koala [15]–[17]. The prevalence of *M. ulcerans* infection in the possum populations in this study cannot be accurately estimated due to the inherent difficulties in sampling from a wild population; however, it appears that CRT possums are significantly more susceptible to clinical *M. ulcerans* infection than other possum species. Male CRT possums appeared to be particularly predisposed to clinical lesions, perhaps due to behaviours such as fighting (which was touted as a possible reason why male koalas appeared over-represented in an earlier study [15]) or as a result of stress associated with competition for territories. More extensive trapping and sampling and/or captive studies would be informative in revealing whether CRT possums have higher susceptibility to infection and disease across all BU-endemic areas in Victoria. Further, it is noteworthy that only CRT possums were observed with advanced clinical disease, with a number of animals displaying multiple lesions at disparate anatomical sites, and that some cases progressed to systemic disease involving the liver and lungs.

It is unclear whether the apparent susceptibility of the CRT possum is a function of impaired immunity or epidemiological factors such as high rates of environmental exposure, perhaps via communal sharing of contaminated dens (CRT possums are more social than most other possum species [40]), inoculation of the bacterium via penetrative wounds or insect vectors, or possibly via the practice of auto and/or allocoprophagy [41]. Due to the economic impact of CBT possums as reservoirs for *M. bovis* in New Zealand, much effort has been expended in assessing the immunobiology of this species. No such data exist for the CRT possum and testing of the immunological competence of non-infected controls is needed to establish whether there are any inherent immunological deficiencies in these populations, and would be helpful in differentiating any local or systemic immunosuppressive effects of mycolactone in infected individuals.

The significance of variable levels of *M. ulcerans* DNA in the gastrointestinal tract of both clinically and subclinically affected possums in endemic areas is unknown. There were no lesions consistent with established gastrointestinal infection in any animals where histopathological examination of the gut was performed. The question as to whether these are viable organisms (and therefore represent a significant source of potentially infective organisms to other animals and people) remains unanswered, despite concerted attempts to culture *M. ulcerans* from fresh and aged PCR-positive faecal material collected from the environment, and fresh gut contents collected at autopsy. A single isolate was recovered from the small intestinal contents of a systemically affected CRT possum from Phillip Island, however the possibility of cross-contamination from lesions cannot be ruled out in this instance. Alternative methods to demonstrate metabolic activity of *M. ulcerans* within possum gut contents/faeces, for example, via rRNA or mycolactone detection remain a possibility, although negative findings may theoretically result if the organisms have become metabolically dormant.

Based on real-time PCR signal strength, there is no evidence that the organism is amplified in any particular region of the gut of affected animals. Nor does it appear that clinically affected cases have significantly greater amounts of *M. ulcerans* DNA in the gut compared to subclinical cases. There is also no sex predilection for PCR-positivity in the gut contents or faeces, perhaps suggesting a common environmental source of oral inoculation in all animals (even though there may be separate risk factors for clinical disease between the sexes).

If the organisms are viable within the gut, the daily practice of autocoprophagy in CRT possums [42] provides a possible route for re-inoculation and amplification of organisms in the gut of an individual (and thus ongoing colonisation/infection). It is not known whether this represents a source of horizontal transmission to other individuals (thus maintaining colonisation/infection in the population) via allocoprophagy. This seems most likely, perhaps, in juveniles approaching weaning. In the koala, which like the CRT is also a specialist folivore, establishment of normal gut flora in dependent juveniles occurs during weaning via allocoprophagy [43]. An alternative significant environmental source of oral inoculation (for example, a contaminated food source) remains elusive, although interrogation of a variety of plant species (potentially foraged by CRT possums in some endemic areas) via IS*2404* real-time PCR demonstrated low levels of *M. ulcerans* DNA (data not shown). Except in one instance, only animals with cutaneous lesions were found to have PCR-positive buccal swabs, which might suggest that the positivity of these samples may be due to contamination of the oral cavity via licking of ulcers. However, it cannot be conclusively shown that these findings are not due to ingestion of *M. ulcerans* DNA from the environment or contaminated caecotrophs.

The fact that the non-coprophagous CBT has also been noted to have similar levels of *M. ulcerans* DNA in the faeces [19] also argues for a potential oral environmental source. It is unknown at this stage whether the finding of *M. ulcerans* DNA in the gut of possums represents transient or persistent gut contamination, colonisation or infection, although in two CBT and four MBT possums trapped repeatedly over the course of the study low level faecal PCR-positivity was observed to be transient. The possibility that the *M. ulcerans* DNA was derived from soil or environmental detritus contamination of the external surface of the faeces was considered unlikely, given that the samples were mostly collected directly from within traps or collection boxes, rather than the ground. No record of whether gut PCR-positivity was transient or permanent in CRT possums was obtained due to the difficulties of repeat trapping of this species, even with the aide of radio-tracking collars (A. Legione, K Handasyde unpublished observations).

It is also not known whether gut colonisation/infection is directly linked with prior or eventual clinical disease. The only clinically affected individual without PCR-positive faeces (case 13) had numerous superficial cutaneous lesions on the tail that were different in appearance from the typically deep, undermined ulcers observed in other animals in the study. Whilst these lesions were weakly PCR-positive, it cannot be excluded that they were actually caused by a different disease entity (such as pox-virus infection [44]) that had become contaminated with *M. ulcerans* DNA from the environment.

Based on real-time PCR results of urine samples, it does not appear that *M. ulcerans* is shed via the urinary tract, nor is there evidence of mycobacteraemia in the animals from which blood samples were obtained. Thus, it is unlikely that possums are a significant source of potentially infective blood meals for vectors such as mosquitoes or flies, although mechanical transmission from the wounds of infected possums to humans via contaminated insect vectors cannot be ruled out.

While the natural history of this disease in possums is generally unknown, lesions were observed to undergo spontaneous remission in a CBT and a MBT possum in this study. It is not known if either of these two individuals eventually suffered disease relapse (one individual was kept in captivity but has been lost to follow-up, and one was released back into the wild). Due to the aforementioned difficulties in repeated trapping of CRT possums it is unknown whether clinical lesions are able to undergo spontaneous remission in this species. Given the state of ill-health of some of the CRT possums in this study, (and possibly the CBT possum, case 28) it seems that the disease may become progressive, possibly leading to death either directly due to the effects of the *M. ulcerans* infection, or due to secondary illness (although no co-morbidities were identified in severely affected animals that underwent autopsy). Unfortunately, in the two animals found deceased (cases 26 and 28) the cause of death could not be determined due to the state of decomposition, thus it is impossible to say what role, if any, *M. ulcerans* played in their demise.

The systemically affected CRT possums had histopathological evidence of disease in the lungs and liver. Lower respiratory tract infections have been previously reported in koalas with extensive nasal cavity disease, presumably due to inhalation of the organisms [17]. The authors speculated that due to the organism's strict temperature requirements (27–33°C) the infection was possibly maintained in this anatomical site due to a sub-normal core body temperature that could occur in a moribund animal. Unfortunately, the core body temperature of the severely affected possums in this study was not measured, however the normal core body temperature of the CRT possum is 35–36°C [45], thus it is conceivable that these individuals may become sufficiently hypothermic to allow growth of *M. ulcerans* within the body [39]. Circulating mycolactone has been detected in human patients [46], and may also play a role in producing systemic immunosuppression in animals with extensive disease.

Our initial study of *M. ulcerans* infection in possums has highlighted a number of areas that warrant further investigation. More detailed studies are necessary to document the natural history of the disease and the level of *M. ulcerans* DNA shed in the faeces over time, in both naturally and experimentally infected animals (especially CRT possums). One difficulty is that the stress of bringing wild possums into captivity is likely to confound results from any such investigations, and although ideally such aims could be achieved via radio-tracking of wild animals, the heavy attrition of CRT possums in their natural habitat [47], makes this work difficult, as a pilot study by our group has confirmed (A. Legione, K. Handasyde Unpublished observations). Research into the potential for horizontal and vertical transmission between possums is also needed, as well as ongoing attempts to determine the viability of *M. ulcerans* within gut contents/faeces.

In conclusion, the disease burden in CRT possums (especially males) in some areas of Victoria endemic for *M. ulcerans* disease appears significant. Whilst it appears that CBT and MBT possums with solitary cutaneous lesions have the ability to overcome the infection, the natural history of the disease generally remains unknown. In some instances, severely affected animals, especially CRT possums, may become systemically, and potentially fatally affected. As previous work has shown, subclinical gut carriage of *M. ulcerans* DNA in possums is quite common [19], and this study has shown that in some CBT and MBT possums it is transient. It is unknown whether this is also the case for CRT possums. Further work is required to establish whether this disease poses a potential threat to possum populations, and whether these animals are contributing to the high incidence of *M. ulcerans* infection in people in certain geographical areas by acting as environmental reservoirs.

## Supporting Information

Figure S1 Box-and-whisker plot of real-time IS*2404* PCR cycle threshold (C_T_) values of faeces collected from clinically and sub-clinically affected possums.
(TIF)
