# Supplementary figures and images for: Clinical, Microbiological and Pathological Findings of Mycobacterium ulcerans Infection in Three Australian Possum Species
Source: PLoS Negl Trop Dis. 2014 Jan 30;8(1):e2666. doi: 10.1371/journal.pntd.0002666 (PMC3907337; doi:10.1371/journal.pntd.0002666)

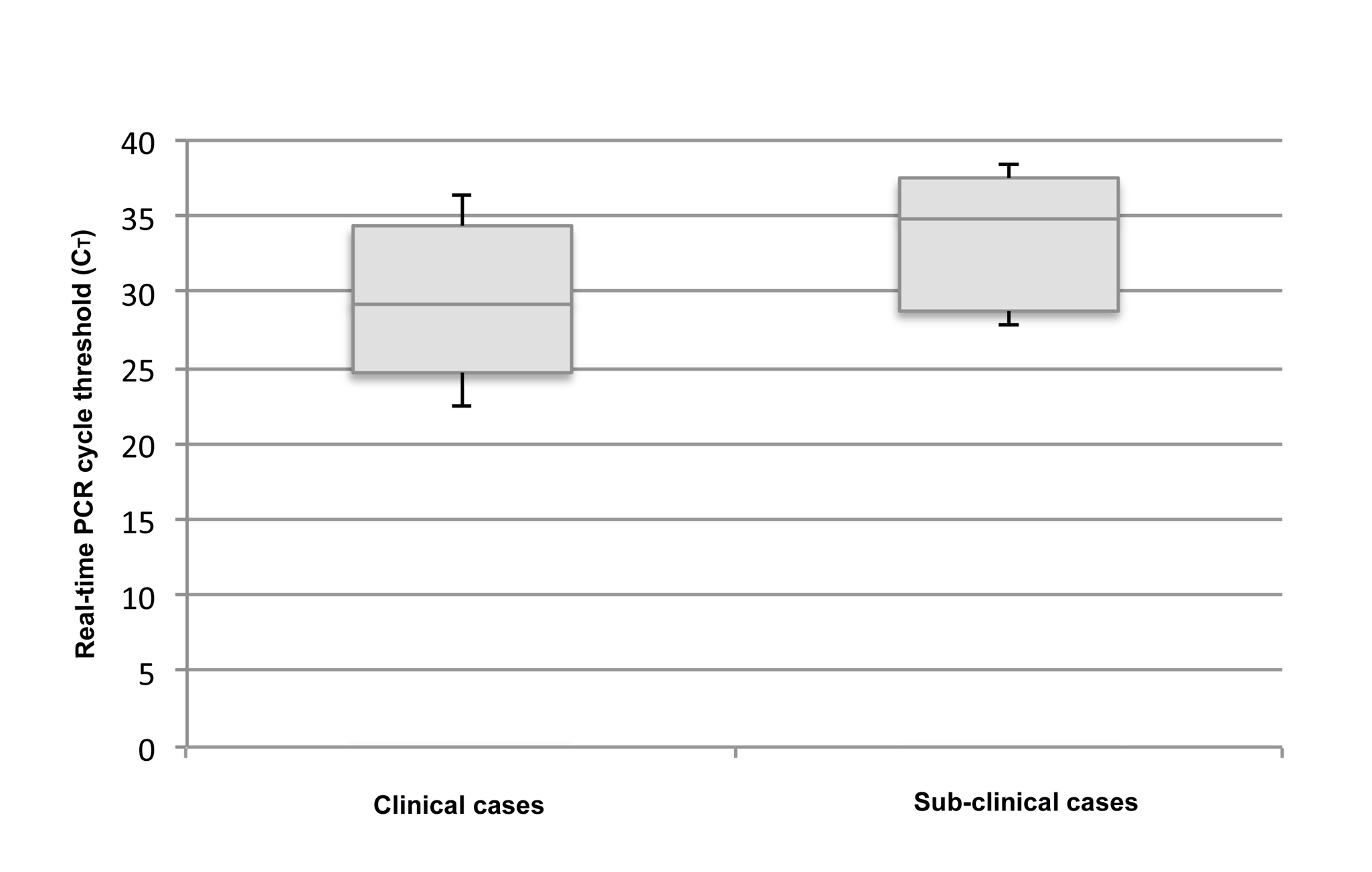

Supplement: Figure S1 — Box-and-whisker plot of real-time IS2404 PCR cycle threshold (CT) values of faeces collected from clinically and sub-clinically affected possums. (TIF) [file pntd.0002666.s001.tif]
